# The efficacy of Qingjin Huazhuo Decoction combined with acupuncture therapy on pulmonary function and quality of life in patients with acute exacerbation of chronic obstructive pulmonary disease

**DOI:** 10.12669/pjms.42.3.13454

**Published:** 2026-03

**Authors:** Yun Liu, Yuzhen Pei, Zenglu Kang, Hongqian Yan, Xiangyan Yu

**Affiliations:** 1 Yun Liu, Department of Respiratory, Hebei Provincial Traditional Chinese Medicine Hospital, Shijiazhuang, Hebei Province 050000, P.R. China; 2 Yuzhen Pei, Department of Respiratory, Hebei Provincial Traditional Chinese Medicine Hospital, Shijiazhuang, Hebei Province 050000, P.R. China; 3 Zenglu Kang, Department of Respiratory, Hebei Provincial Traditional Chinese Medicine Hospital, Shijiazhuang, Hebei Province 050000, P.R. China; 4 Hongqian Yan, Department of Respiratory, Hebei Provincial Traditional Chinese Medicine Hospital, Shijiazhuang, Hebei Province 050000, P.R. China; 5 Xiangyan Yu, Department of Respiratory, Hebei Provincial Traditional Chinese Medicine Hospital, Shijiazhuang, Hebei Province 050000, P.R. China

**Keywords:** Acupuncture therapy, AECOPD, Pulmonary function, Qingjin Huazhuo Decoction, Quality of life

## Abstract

**Objective:**

To evaluate the efficacy of Qingjin Huazhuo Decoction combined with acupuncture in patients with acute exacerbation of chronic obstructive pulmonary disease (AECOPD).

**Methodology:**

A retrospective analysis was conducted on 80 AECOPD patients admitted between October 2023 and February 2025. Patients were divided into a control group (n=40, treated with standard Western medicine) and a study group (n=40, treated with standard therapy plus Qingjin Huazhuo Decoction and acupuncture). Pulmonary function, clinical symptoms, exercise capacity, inflammatory markers, blood gas parameters, and adverse events were compared before and after treatment.

**Results:**

After treatment, both groups showed improvements in pulmonary function indicators (FEV1, FVC, PEF, FEV1/FVC), with the study group showing significantly greater gains (P<0.05). The study group also had greater improvements in mMRC and CAT scores, and longer 6-minute walk distance. Serum IL-6 and CRP levels declined significantly in both groups, with larger reductions observed in the study group (P<0.05). Post-treatment PaCO_2_ was lower and PaO_2_ higher in the study group compared to controls (P<0.05). No significant difference was found in the incidence of adverse reactions between the groups.

**Conclusion:**

In this retrospective analysis, Qingjin Huazhuo Decoction combined with acupuncture therapy was associated with improved pulmonary function, exercise capacity, symptom/health status, inflammatory markers, and blood gas parameters in hospitalized patients with AECOPD, with comparable safety to conventional therapy. These findings are hypothesis-generating and should be confirmed in prospective randomized controlled trials.

## INTRODUCTION

According to data from the Global Burden of Disease Study, chronic obstructive pulmonary disease (COPD) has become the third leading cause of death worldwide.^1^ Acute exacerbation of COPD (AECOPD) often leads to accelerated decline in patients’ lung function, markedly lowering the quality of life, and increasing mortality.^2^-^4^ The mortality rate of patients within one year after an acute exacerbation can rise to 20% - 40%, and each exacerbation may cause irreversible damage to lung function.^5^-^7^

Although Western medicine treatment for AECOPD, which mainly includes bronchodilators, glucocorticoids, and anti-infective drugs, can quickly relieve airway spasm and inflammation, its effect on the quality of life is limited, and long-term application is linked to adverse reactions such as drug resistance and osteoporosis.^8^,^9^ In traditional Chinese medicine (TCM), AECOPD is classified into the categories of “dyspnea syndrome” and “lung distension”. It is believed that the core pathogenesis of the disease lies in the failure of the lung to disperse and descend, phlegm turbidity blocking the lung, and qi stagnation and blood stasis.

The treatment needs to follow the basic principles of clearing heat and resolving phlegm, and diffusing the lung to reduce turbidity.^10^ Qingjin Huazhuo Decoction is a classic prescription based on the above theory, composed of Scutellaria baicalensis, Morus alba bark, and other medicines. It has the effects of clearing heat and moistening the lung, resolving phlegm, and dissipating binds. Modern pharmacological studies have confirmed that it can inhibit the release of airway inflammatory factors and reduce excessive mucus secretion.^11^ Acupuncture therapy regulates the functions of the lung, spleen, and kidney by stimulating acupoints such as Feishu (BL13), Tanzhong (CV17), and Zusanli (ST36), and enhances airway clearance ability. Studies have shown that it can improve respiratory function in COPD patients.^12^

However, there are few studies on the application of Qingjin Huazhuo Decoction combined with acupuncture therapy in patients with AECOPD. Furthermore, the synergistic effect of the combined treatment on the lung function and quality of life has not been systematically verified. This retrospective study aimed to evaluate the clinical efficacy of Qingjin Huazhuo Decoction combined with acupuncture therapy in AECOPD patients, and to provide evidence-based medical basis for optimizing AECOPD treatment strategies.

## METHODOLOGY

A total of 80 AECOPD patients admitted to Hebei Provincial Traditional Chinese Medicine Hospital from October 2023 to February 2025 were retrospectively selected and grouped based on the treatment regimen: 40 patients who received conventional western medicine treatment comprised the control group, and 40 patients who received traditional western medicine combined with TCM treatment (Qingjin Huazhuo Decoction combined with acupuncture therapy) were assigned to the study group.

### Ethical approval:

This study was approved by the Ethics Committee of Hebei Provincial Traditional Chinese Medicine Hospital (Approval number: HBZY2022-KY-054-01; Date: September 20, 2022).

### Inclusion criteria:

TCM diagnosis conforms to the diagnostic criteria for AECOPD in International Clinical Practice Guidelines for TCM: Chronic Obstructive Pulmonary Disease,^13^ including:

1. Cough or dyspnea with wheezing.
2. Profuse phlegm, yellow or white sticky in color, with difficulty in expectoration.
3. Fever or thirst with preference for cold drinks.
4. constipation.
5. Red tongue, yellow or yellow greasy coating, or rapid or slippery and rapid pulse.

The diagnosis can be confirmed if two items from One and Two are met, plus two items from three, four and five. Western medicine diagnosis conforms to the diagnostic criteria for COPD;^14^ the disease is in the acute exacerbation phase (with increased cough, sputum production, wheezing, increased sputum volume or purulent sputum, with or without fever).

- Aged 18-80 years.
- Pulmonary function tests indicate that forced expiratory volume in one second (FEV1) and forced vital capacity (FVC) ratio (FEV_1_/FVC) < 70% after inhalation of bronchodilators, and FEV_1_% predicted (FEV_1_%pred) is 30%-80%.
- The duration of acute exacerbation is ≤ 7 days, and no systematic treatment for this exacerbation phase (such as systemic glucocorticoids, antibiotics) has been received.
- Able to cooperate with pulmonary function tests, scale scoring, and follow-up.
- Patients or their family members have signed the informed consent form and voluntarily participated in this study.

### Exclusion criteria:

- Complicated with other severe lung diseases such as bronchial asthma, pulmonary tuberculosis, pulmonary embolism, or lung cancer.
- Having severe functional failure of essential organs such as the heart, liver, or kidney (such as heart failure with NYHA class IV decompensated liver cirrhosis, uremic stage of renal insufficiency).
- Having a history of mental illness, cognitive impairment, or being unable to communicate and cooperate.
- Having definite allergies or contraindications to the components of Qingjin Huazhuo Decoction or acupuncture therapy (such as coagulation dysfunction, local skin infection).
- Participated in other similar clinical trials within the past three months.
- Pregnant or lactating women.
- Having malignant tumors, immunodeficiency diseases, or receiving immunosuppressant therapy.

### Treatment schemes:

### Conventional Western medicine treatment:

- oAnti-infective treatment: specific antibiotics were selected according to the results of patients’ sputum culture and drug sensitivity test, and the dosage was adjusted according to the condition.
- oAnti-asthmatic treatment: Seretide 50/250 was given, one inhalation each time, twice a day. For patients with severe conditions, methylprednisolone (40-80mg/day, intravenous drip) was added, and the dosage was gradually reduced until withdrawal after symptoms were relieved.
- oExpectorant treatment: Acetylcysteine effervescent tablets (600mg each time, twice a day) or ambroxol oral liquid (30mg each time, three times a day) were taken orally to promote sputum dilution and excretion.
- oBasic supportive treatment, including continuous low-flow oxygen inhalation (oxygen flow rate 1-2L/min, maintaining blood oxygen saturation at 90%-92%), nutritional support, and symptomatic treatment (such as reducing fever, correcting water-electrolyte disorders). Arterial blood gas sampling was performed while patients were receiving this stable low-flow oxygen supplementation (1–2 L/min via nasal cannula), and no noninvasive ventilation (NIV) was used during the treatment period or at the time of sampling.

### TCM treatment with Qingjin Huazhuo Decoction and acupuncture:

The regimen was given on the basis of the traditional Western treatment (described above) and included:

### Qingjin Huazhuo Decoction:

Cortex Mori 12g, stir-fried Fructus Perillae 10g, Fructus Trichosanthis 10g, Pericarpium Citri Reticulatae 10g, Rhizoma Pinelliae Preparatum 9g, Poria cocos 15g, Radix Scutellariae 9g, Arisaema Cum Bile 9g, Rhizoma Zingiberis Recens 10g, Radix Glycyrrhizae 6g, Periostracum Cicadae 10g, Semen Armeniacae Amarum 6g. All crude herbs were dispensed by the hospital pharmacy and were purchased from certified suppliers with traceable batch numbers, in accordance with the standards of the Chinese Pharmacopoeia. Upon receipt, the pharmacy performed routine quality checks (including appearance inspection and batch-to-batch consistency verification) before use. The decoction was prepared centrally by the hospital pharmacy using a unified protocol to minimize inter-patient variability: one daily dose was soaked in 500 mL of purified water for 30 minutes, brought to a boil over high heat, then simmered for 30 minutes; the final filtrate was approximately 200 mL and was divided into two portions. Patients were instructed to take the decoction warm twice daily at fixed times (morning and evening) throughout the two weeks treatment period.

### Acupuncture therapy:

Acupoints selected were Tanzhong (CV17), bilateral Feishu (BL13), bilateral Zusanli (ST36), bilateral Tianshu (ST25), bilateral Dingchuan (EX-B1), bilateral Taiyuan (LU9), bilateral Daheng (SP15), Zhongwan (CV12), and bilateral Fenglong (ST40). Patients took a supine or prone position. After routine disinfection of the acupoint area, a filiform needle of 0.30mm × 40mm was used. All acupuncture sessions were performed by the same senior TCM practitioner (attending physician) with more than 10 years of clinical acupuncture experience, following a standardized operating procedure to minimize operator-related variability. Tanzhong was needled horizontally 0.5-0.8 cun; Feishu was needled obliquely 0.5-0.8 cun; Zusanli was needled straightly 1.0-1.5 cun; Tianshu was needled straightly 1.0-1.5 cun; Dingchuan was needled straightly 0.5-0.8 cun; Taiyuan was needled straightly 0.3-0.5 cun; Daheng was needled straightly 1.0-1.5 cun; Zhongwan was needled straightly 1.0-1.5 cun; Fenglong was needled straightly 1.0-1.5 cun. After obtaining qi, the even reinforcing-reducing method was adopted, and the needle was retained for 30 minutes. Deqi was defined as the patient’s subjective sensation of soreness, numbness, distention, or heaviness at the needling site and was consistently elicited prior to needle retention. The treatment was performed once a day. Both groups were treated for two weeks.

### Treatment adherence and completion:

As this was a retrospective study based on inpatient medical records, administration of Qingjin Huazhuo Decoction and delivery of acupuncture sessions were supervised and documented by trained hospital staff during hospitalization. All 80 patients included in the final analysis completed the full two week treatment course, and no premature discontinuation or loss to follow-up occurred during the intervention period.

### Observation indicator:

The primary outcomes were changes in pulmonary function parameters (PEF, FEV1, FVC, and FEV1/FVC) from baseline to the end of the two week treatment period, as these measures objectively reflect airflow limitation and are widely used to evaluate treatment response in COPD. Secondary outcomes included symptom burden and health status (mMRC and CAT), exercise capacity (6MWD), systemic inflammation (serum IL-6 and CRP), blood gas parameters (PaO_2_ and PaCO_2_), and adverse events, which were assessed as supportive endpoints to provide a comprehensive evaluation of clinical benefit and safety. The following indicators were collected from all patients:

- The pulmonary function of the two groups before and after treatment. Peak expiratory flow (PEF), FEV1, FVC, and FEV1/FVC were detected by a pulmonary function instrument.
- The body function recovery of the two groups before and after treatment, including quality of life, disease severity, and exercise capacity. The disease severity was evaluated by the modified Medical Research Council dyspnea scale (mMRC), with scores ranging from 0 to 4 points, and lower scores indicating better conditions. The quality of life was evaluated by the COPD Assessment Test (CAT), with a total score of 40 points, and lower scores indicating better conditions. The exercise capacity was assessed by the 6-minute walk distance test (6MWD).
- The serum levels of inflammatory factors (IL-6 and CRP) in the two groups before and after treatment were measured in 4ml of fasting venous blood by enzyme-linked immunosorbent assay.
- The blood gas status of the two groups before and after treatment, including partial pressure of carbon dioxide (PaCO_2_) and partial pressure of oxygen (PaO_2_), was determined using an automatic blood gas analyzer; all samples were collected under a consistent, stable low-flow oxygen supplementation regimen (1–2 L/min via nasal cannula, target SpO_2_ 90%–92%), and no noninvasive ventilation was applied during sampling.
- The adverse reactions of the two groups were statistically analyzed.

### Statistical analysis:

All analyses were performed using SPSS version 26.0 (IBM Corp., Armonk, NY, USA). Continuous variables are presented as mean ± standard deviation (SD) and categorical variables as number (percentage). Prior to hypothesis testing, normality was assessed using the Shapiro–Wilk test and homogeneity of variance was evaluated using Levene’s test. For between-group comparisons, independent-samples t-tests were used when assumptions were met; for within-group (pre–post) comparisons, paired-samples t-tests were applied. Categorical variables were compared using the chi-square test. All tests were two-sided, and a P value < 0.05 was considered statistically significant.

## RESULTS

There were no statistically significant differences in general data such as gender, age, duration of acute exacerbation, GOLD stage, body mass index, smoking history, and drinking history between the two groups (P > 0.05) (Table-I).

**Table-I:** Comparison of General Data Between the Two Groups.

Items	Study Group (n = 40)	Control Group (n = 40)	t/χ²	P
***Gender [n (%)]***				
Male	26(65.00)	23(57.50)	0.474	0.491
Female	14(35.00)	17(42.50)		
Age(x̅±*s*, years old)	50.08±8.62	48.69±9.44	0.688	0.494
Duration of Acute Exacerbation(x̅±*s*, d)	3.89±1.63	4.02±1.38	0.385	0.701
***GOLD Stage [n (%)]***				
Stage II	13(32.50)	11(27.50)	0.471	0.790
Stage III	16(40.00)	19(47.50)		
Stage IV	11(27.50)	10(25.00)		
Body Mass Index(x̅±*s*, kg/m^2^)	23.13±2.08	22.83±2.24	0.621	0.537
***Smoking History [n (%)]***				
Yes	25(62.50)	22(55.00)	0.464	0.496
No	15(37.50)	18(45.00)		
***Drinking history [n (%)]***				
Yes	27(67.50)	29(72.50)	0.238	0.626
No	13(32.50)	11(27.50)		

Before treatment, PEF, FEV1, FVC, and FEV1/FVC were comparable in the two groups (P > 0.05). Table-II After treatment, PEF, FEV1, FVC, and FEV1/FVC in both groups increased, and were significantly higher in the study group compared to the control group (P < 0.05).

**Table-II:** Comparison of Pulmonary Function Between the Two Groups(*x̅*±*s*).

Time	Group	Number of Cases	PEF(L/min)	FEV1(L)	FVC(L)	FEV1/FVC (%)
Before Treatment	Study Group	40	378.53±12.25	1.39±0.35	2.71±0.59	51.29±5.88
	Control Group	40	380.21±10.34	1.42±0.31	2.67±0.64	53.18±6.02
	*t*		0.663	0.406	0.291	1.420
	*P*		0.509	0.686	0.772	0.160
After Treatment	Study Group	40	519.18±16.20	1.98±0.47	3.41±0.45	58.06±3.51
	Control Group	40	490.52±14.61	1.75±0.42	3.10±0.37	56.45±3.60
	*t*		8.309	2.308	3.365	2.025
	*P*		0.000	0.024	0.001	0.046

Similarly**,** pretreatment mMRC, CAT, and 6MWD scores of both groups were similar (P > 0.05). After the treatment, the mMRC and CAT scores in both groups significantly decreased, and the 6MWD score increased. The mMRC and CAT scores of the study group were lower than those of the control group, while the 6MWD was higher (P < 0.05). Table-III,

**Table-III:** Comparison of Functional Rehabilitation Effects Between the Two Groups (*x̅*±*s*).

Time	Group	Number of Cases	mMRC(Score)	CAT(Score)	6MWD(m)
Before Treatment	Study Group	40	2.60±0.68	20.24±5.08	331.44±47.12
	Control Group	40	2.58±0.71	19.57±4.86	329.67±50.04
	*t*		0.129	0.603	0.163
	*P*		0.898	0.548	0.871
After Treatment	Study Group	40	1.79±0.24	8.02±1.94	489.50±52.20
	Control Group	40	2.16±0.28	9.95±2.57	461.13±47.37
	*t*		6.345	3.791	2.545
	*P*		0.000	0.000	0.013

Serum levels of IL-6 and CRP were comparable in the two groups before the treatment (P > 0.05). Both treatment approaches led to a considerable decrease in the levels of inflammatory factors. Post-treatment levels of serum IL-6 and CRP in patients in the study group were significantly lower than in the control group (P < 0.05; Table-IV).

**Table-IV:** Comparison of Inflammatory Factors Between the Two Groups(*x̅*±*s*).

Time	Group	Number of Cases	IL-6(ng/L)	CRP(mg/L)
Before Treatment	Study Group	40	17.53±3.19	19.64±5.25
	Control Group	40	16.98±4.02	20.05±6.13
	*t*		0.678	0.321
	*P*		0.500	0.749
After Treatment	Study Group	40	5.59±1.74	5.57±1.27
	Control Group	40	7.21±2.18	7.95±2.15
	*t*		3.673	6.028
	*P*		0.000	0.000

In terms of the blood gas status, both groups demonstrated similar pretreatment PaCO_2_ and PaO_2_ (P > 0.05). As shown in Table-V, after the treatment, PaCO_2_ in both groups decreased, and PaO_2_ increased compared to pre-treatment levels. The effect was more prominent in the study group than in the control group (P < 0.05). As summarized in Table-VI, there was no significant difference in the incidence of adverse reactions between the study group (10.00%) and the control group (5.00%) (P > 0.05).

**Table-V:** Comparison of Blood Gas Status Between the Two Groups (*x̅*±*s*, mmHg).

Time	Group	Number of Cases	PaCO_2_	PaO_2_
Before Treatment	Study Group	40	63.26±6.68	61.17±8.37
	Control Group	40	61.97±7.08	60.66±9.25
	*t*		0.838	0.259
	*P*		0.405	0.797
After Treatment	Study Group	40	35.44±3.39	96.21±3.41
	Control Group	40	37.55±4.26	92.18±3.30
	*t*		2.451	5.371
	*P*		0.017	0.000

**Table-VI:** Comparison of Adverse Reactions Between the Two Groups [n(%)].

Group	Number of Cases	Rash	Fatigue	Palpitation	Nausea and Vomiting	Total Incidence
Study Group	40	1(2.50)	0(0.00)	2(5.00)	1(2.50)	4(10.00)
Control Group	40	0(0.00)	1(2.50)	0(0.00)	1(2.50)	2(5.00)
*χ* ^2^						0.180
*P*						0.671

## DISCUSSION

This study explored the implementation of the integrated TCM and Western medicine regimen in the treatment of AECOPD by comparing the efficacy differences between conventional Western medicine treatment and the combination of Qingjin Huazhuo Decoction and acupuncture therapy. The results showed that the combined treatment was significantly superior to Western medicine alone in improving pulmonary function, enhancing functional rehabilitation, reducing inflammatory responses, and optimizing blood gas status, with comparable safety, providing a better option for the clinical treatment of AECOPD. However, because this was a retrospective, non-randomized observational comparison, the findings should be interpreted as associative and hypothesis-generating, rather than confirmatory evidence of causality.

In terms of improving pulmonary function, the study group had significantly higher PEF, FEV_1_, FVC, and FEV_1_/FVC than the control group after treatment (P < 0.05), confirming that Qingjin Huazhuo Decoction combined with acupuncture therapy can more effectively enhance airway ventilation function. From the perspective of traditional Chinese medicine (TCM), AECOPD is characterized by “phlegm-heat obstructing the lung” and “failure of the lung to disperse and descend”. From a contemporary biomedical viewpoint, these concepts can be understood as corresponding to acute airway inflammation, mucus hypersecretion with impaired mucociliary clearance, and consequent airflow limitation and ventilation–perfusion imbalance that collectively aggravate dyspnea and gas-exchange impairment during exacerbations.^15^ In Qingjin Huazhuo Decoction, Cortex Mori and Radix Scutellariae clear lung fire, targeting the “heat” pathogen; Fructus Trichosanthis, Arisaema Cum Bile, and Rhizoma Pinelliae Preparatum resolve phlegm and dissipate binds, directly addressing “phlegm” turbidity; stir-fried Fructus Perillae and Semen Armeniacae Amarum descend qi and relieve asthma, restoring the lung’s depurative and descending function; Poria cocos and Pericarpium Citri Reticulatae strengthen the spleen and resolve dampness, reducing phlegm production based on the theory that “the spleen is the source of phlegm production”.

The entire formula works together to clear heat, resolve phlegm, diffuse the lung, and reduce turbidity. This TCM framework provides a clinically intuitive description of the exacerbation phenotype, in which “heat” and “phlegm” manifest as intensified inflammatory activity and excessive, viscous sputum in the airways. Therefore, interventions aimed at “clearing heat” and “resolving phlegm” may be translated into modern terms as attenuating pro-inflammatory signaling, reducing mucus burden, and improving airway patency, thereby facilitating recovery of ventilatory function.^16^ Modern pharmacological studies have shown that the moracins in Cortex Mori can relax smooth bronchial muscles; baicalin in Radix Scutellariae can inhibit the release of airway inflammatory factors (such as IL-6); and amygdalin in Semen Armeniacae Amarum can improve airway mucociliary clearance function, all of which provide a material basis for the improvement of pulmonary function indicators.^17^ In acupuncture therapy, Feishu (BL13) and Dingchuan (EX-B1) can regulate lung ventilation through nerve reflexes; Taiyuan (LU9), as the source point of the Lung Meridian, can tonify lung qi and unblock meridians, while Tanzhong (CV17), known as the “gathering point of qi”, can broaden the chest and regulate qi. These acupoints synergize with drugs to enhance the lung’s dispersing and descending abilities, collectively improving airflow limitation.^18^ A study by Chen W et al.^19^ also confirmed that phlegm-resolving and turbidity-reducing formulas combined with acupuncture can significantly improve pulmonary function and other indicators in COPD patients, which is consistent with the results of this study. Beyond segmental reflex regulation, acupuncture may exert systemic effects through the neuroimmune–endocrine network, including modulation of autonomic balance and inflammatory tone. In particular, stimulation-induced afferent signaling may engage vagal pathways and stress-response circuitry (such as hypothalamic–pituitary–adrenal axis), which has been proposed to contribute to downregulation of pro-inflammatory mediators and to improved respiratory muscle coordination, thereby complementing the anti-inflammatory and airway-clearance effects of the herbal formula.^12^,^18^,^20^,^21^

In terms of functional rehabilitation and quality of life, the study group had lower mMRC and CAT scores and a longer 6MWD (P < 0.05), indicating that the combined treatment has more advantages in relieving symptoms, improving quality of life, and enhancing activity endurance. Importantly, the magnitude of change observed in the study group appears to be clinically meaningful rather than merely statistically significant. Specifically, 6MWD increased from 331.44 ± 47.12 m to 489.50 ± 52.20 m (an approximate gain of 158 m), which exceeds the commonly reported minimal clinically important difference (MCID) for COPD (approximately 30–54 m).^22^,^23^ In parallel, the mMRC score decreased from 2.60 ± 0.68 to 1.79 ± 0.24 and the CAT score decreased from 20.24 ± 5.08 to 8.02 ± 1.94, both surpassing widely accepted MCID thresholds (≥1 point for mMRC and ≥2 points for CAT), suggesting substantial improvement in perceived breathlessness and health-related quality of life.^24^ These improvements are particularly noteworthy in hospitalized patients during the acute exacerbation phase, when functional limitation and activity intolerance are typically prominent, supporting the real-world feasibility of the combined regimen for accelerating inpatient recovery.^5^,^14^ The dyspnea and decreased quality of life in AECOPD patients are mainly related to airway obstruction and physical insufficiency. Qingjin Huazhuo Decoction quickly relieves symptoms such as cough, sputum production, and wheezing, reducing the interference of symptoms on daily activities; acupuncture at Zusanli (ST36), Zhongwan (CV12), and other acupoints can invigorate the spleen and replenish qi, improving skeletal muscle energy metabolism and enhancing exercise endurance (manifesting in the extension of 6MWD) based on the theory that “the spleen governs the limbs”.

Meanwhile, the improvement in dimensions such as “limited daily activities” and “emotional impact” in the CAT score stems not only from the relief of physical symptoms but also from the enhanced confidence of patients due to disease control. Huang L et al.^25^ found that the integrated TCM and Western medicine regimen can reduce the CAT score and extend the 6MWD in COPD patients, which is consistent with the results of this study, further confirming the value of combined treatment in functional rehabilitation.

Changes in inflammatory factor levels provide molecular evidence for the effectiveness of combined treatment. The decrease in IL-6 and CRP in the study group was significantly greater than that in the control group (P < 0.05), indicating a more potent anti-inflammatory effect. A study by Barnes PJ et al.^26^ confirmed that the core mechanism of acute exacerbation of AECOPD is the explosive enhancement of airway inflammation. As key pro-inflammatory factors, IL-6 and CRP are directly involved in airway mucosal damage and excessive mucus secretion.^27^ In Qingjin Huazhuo Decoction, baicalin from Radix Scutellariae can reduce the synthesis of inflammatory factors by inhibiting the NF-κB signaling pathway; chitin from Periostracum Cicadae can regulate immune balance and reduce excessive inflammatory responses; and glycyrrhizic acid from Radix Glycyrrhizae enhances the anti-inflammatory synergistic effect. In acupuncture therapy, Tianshu (ST25) and Daheng (SP15) regulate intestinal flora based on the theory that “the lung and large intestine are interior-exteriorly related”, reducing endotoxin release and indirectly inhibiting systemic inflammatory responses, forming an anti-inflammatory synergy with drugs.

The combined approach also demonstrated its superiority in terms of improving blood gas status. The study group reported higher post-intervention PaO_2_ and lower PaCO_2_ (P < 0.05), reflecting the optimization of gas exchange function by the combined treatment. This effect may be partly due to the improvement in ventilation efficiency after enhanced pulmonary function, and partly related to the synergistic effect of Qingjin Huazhuo Decoction in “resolving phlegm to unblock lung collaterals” and acupuncture therapy in “diffusing the lung to aid gas exchange”. After phlegm turbidity is dissipated, airway resistance decreases, and the ventilation/perfusion ratio becomes more reasonable, which, combined with low-flow oxygen inhalation, can effectively correct hypoxia.

At the same time, enhanced respiratory muscle strength (such as acupuncture at Zusanli (ST36) to strengthen diaphragmatic contractility) promotes carbon dioxide excretion and relieves retention. Li N et al.^28^ found that the integrated traditional Chinese and Western medicine regimen can more effectively improve blood gas indicators in patients with acute exacerbation of COPD, which is consistent with the conclusion of this study, confirming the value of combined treatment in reducing the risk of respiratory failure.

In terms of safety, there was no significant difference in the incidence of adverse reactions between the two groups (P > 0.05), indicating that the combined treatment has good tolerance. In Qingjin Huazhuo Decoction, Rhizoma Zingiberis Recens can moderate the irritation of Rhizoma Pinelliae and Arisaema Cum Bile, reducing gastrointestinal reactions; acupuncture selects conventional acupoints such as Feishu (BL13) and Zusanli (ST36), with a low risk of fainting or hematoma when operated standardly, providing a safety guarantee for clinical promotion.

### Strengths of the study:

Strengths of the present study include its pragmatic real-world inpatient setting and standardized delivery of an integrative regimen combining Qingjin Huazhuo Decoction and acupuncture on top of conventional care. The study also employed a multidimensional evaluation framework—covering pulmonary function as the primary endpoint, symptom/health status scores, exercise capacity, inflammatory biomarkers, arterial blood gas parameters, and safety outcomes—which provides a more comprehensive picture of clinical benefit during the acute hospitalization phase. Moreover, because both herbal administration and acupuncture sessions were implemented under inpatient supervision, intervention implementation was relatively consistent, supporting the feasibility of this combined approach in routine ward-based AECOPD management.

To confirm the observed associations and improve the level of evidence, future studies should adopt prospective, multi-center randomized controlled trial (RCT) designs with predefined primary and secondary endpoints and standardized background management. Long-term follow-up (≥6 months) is warranted to evaluate the durability of benefit and clinically meaningful outcomes, including re-exacerbation frequency, readmission rates, and longitudinal trajectories of pulmonary function and health status. Mechanistic investigations are also needed to clarify how the combined regimen modulates AECOPD pathophysiology; these may include cytokine profiling, immune-pathway mapping, and multi-omics approaches (such as transcriptomics/proteomics) to explore inflammatory signaling, mucus hypersecretion/airway remodeling pathways, and potential neuroimmune interactions related to acupuncture. In addition, integrating TCM syndrome differentiation with biomarker-based phenotyping may facilitate more individualized and translatable integrative treatment strategies.

### Limitations:

This was a single-center, retrospective, non-randomized observational study, and therefore remains susceptible to selection bias and residual confounding. Although baseline characteristics were broadly comparable between groups, several clinically important variables were not systematically captured or controlled, including prior exacerbation history, baseline maintenance medication use, comorbidity burden, and detailed indices of disease severity at admission. Accordingly, the observed associations should be considered hypothesis-generating rather than confirmatory, and causal effects of the combined therapy cannot be established. The external generalizability may also be limited. In particular, our inclusion criteria incorporated traditional Chinese medicine (TCM) syndrome differentiation and all interventions were delivered under standardized inpatient management, which may not fully represent AECOPD populations in other regions, healthcare systems, or outpatient settings where patient characteristics, clinical workflows, and acceptance of TCM interventions can differ. From a statistical perspective, no a priori sample size calculation or power analysis was performed because the sample size was determined by the number of eligible cases available during the study period. Therefore, the study may be underpowered to detect smaller but clinically meaningful effects—especially for secondary outcomes—and some estimates may be unstable. In addition, multiple outcomes were tested without formal adjustment for multiple comparisons, which may increase the risk of Type-I error; thus, statistically significant findings should be interpreted with caution. Although inpatient management likely supported relatively high adherence, we did not apply a standardized quantitative tool to measure adherence to herbal medication intake or acupuncture session completion, which may affect the robustness of outcome interpretation. Finally, mechanistic endpoints were not investigated (such as molecular pathways related to airway mucus secretion), and follow-up was limited; consequently, long-term outcomes such as re-exacerbation frequency and readmission risk could not be evaluated.

## CONCLUSION

In this retrospective cohort, Qingjin Huazhuo Decoction combined with acupuncture therapy was associated with improvements in pulmonary function, symptom burden/health status, inflammatory markers, and arterial blood gas parameters in hospitalized patients with AECOPD, with no evident increase in adverse reactions compared with conventional therapy alone. Given the observational design and potential residual confounding, these findings should be interpreted cautiously and cannot establish causality. Prospective, adequately powered, multi-center randomized controlled trials with predefined endpoints are warranted to confirm efficacy, safety, and generalizability.

### Authors’ contributions:

**YL:** Study design, literature search and manuscript writing.

**YP, ZK, HY and XY:** Data collection, data analysis and interpretation. Critical review

**YL:** was involved in the manuscript revision and validation and is responsible for the integrity of the study. All authors have read and approved the final manuscript.
